# Polydatin Modulates Inflammatory Cytokine Expression in Lipoteichoic Acid-Stimulated Human Dental-Pulp Stem Cells

**DOI:** 10.3390/jfb16090331

**Published:** 2025-09-05

**Authors:** Rawan Al-Ateeq, Mona Elsafadi, Manikandan Muthurangan, Solaiman Al-Hadlaq

**Affiliations:** 1 Department of Restorative Dental Sciences, College of Dentistry, King Saud University, Riyadh 12372, Saudi Arabia; 439203957@student.ksu.sa; 2 Stem Cell Unit, Department of Anatomy, College of Medicine, King Saud University, Riyadh 2925, Saudi Arabia; melsafadi@ksu.edu.sa (M.E.); mrangan@ksu.edu.sa (M.M.)

**Keywords:** polydatin, inflammation, lipoteichoic acid, dental pulp, anti-inflammatory, stem cells

## Abstract

Gram-positive bacteria are responsible for initiating dental caries. In this process, lipoteichoic acid (LTA), which is expressed on Gram-positive bacteria cell walls, binds to the dental pulp cells, triggering an immune response, followed by inflammation and eventually pulp necrosis. Polydatin is a polyphenolic compound that has been shown to modulate inflammatory mediators in a manner favorable to healing. The purpose of this study was to assess levels of expression of the most prevalent cytokines in the inflamed pulp after polydatin treatment of LTA-stimulated human dental-pulp stem cells (hDPSCs). LTA-stimulated hDPSCs were treated with polydatin in three different concentrations (0.01 µM, 0.1 µM, and 1 µM). Interleukin-6 (IL-6), interleukin-8 (IL-8), interleukin-10 (IL-10), and tumor necrosis factor-α (TNF-α) levels were measured using reverse transcription–quantitative polymerase chain reaction (RT-qPCR) and enzyme-linked immunosorbent assay (ELISA) were quantified. Treatment with all concentrations of polydatin significantly decreased IL-6 and TNF-α levels as evaluated by ELISA and RT-qPCR, respectively. In addition, a significant reduction was observed in IL-8 levels of mRNA and in ELISA, with 0.01 µM and with 1 µM of polydatin in RT-qPCR. On the other hand, IL-10 levels increased with all of the concentrations. In conclusion, polydatin treatment of LTA-stimulated hDPSCs modulated inflammatory cytokine production by suppressing IL-6, IL-8, and TNF-α levels while elevating IL-10 levels.

## 1. Introduction

Dental caries is the most prevalent oral disease, affecting two billion individuals globally. Although it is a preventable disease with known risk factors that include a diet with high sugar content, poor oral hygiene, inaccessibility to healthcare institutions, and many other risks affecting oral health, it still affects increasing numbers of individuals [1]. The negative impact of oral diseases extends to the general health status, causing pain and loss of function; it drains governmental and individual financial resources and disrupts daily function in society [1]. The dental pulp consists of loose mesenchymal tissue encapsulated within rigid structures consisting of enamel, dentin, and cementum, if these rigid structures are lost or compromised due to mechanical or chemical factors, the pulp will be exposed to the oral environment [2]. The etiologic agent of dental caries is acid-producing microorganisms present in biofilms covering the tooth surface that demineralize hard tooth structure, leading to pulp inflammation and, eventually, pulp exposure and pulp necrosis [2]. The initiation and progression of the carious process are mainly caused by Gram-positive and facultative anaerobic bacteria inhabiting the dental biofilm [3,4]. Dental-pulp cells respond to dental caries microorganisms and their byproducts by activating their immuno-protective role through pathogen-recognition receptors, including NOD-like receptors as well as toll-like receptors expressed on macrophages, dendritic cells, and dental-pulp stem cells (DPSCs) [5,6]. Odontoblasts are the first cells in the dental pulp that encounter microorganisms and their by-products due to their location at the periphery of the dental pulp. While the dental-pulp stem cells are considered a minority, they migrate and react rapidly to the inflamed part of the dental pulp [7]. Among the toll-like receptor family is toll-like receptor-2, which detects and adheres to lipoteichoic acid (LTA), an endotoxin and TLR-2 agonist of the Gram-positive bacteria cell wall, triggering a cascade of immune responses by promoting the secretion of pro-inflammatory and anti-inflammatory cytokines that impact the intensity of the inflammatory process [5,6,8].

The binding of LTA to toll-like receptor-2 in DPSCs activates the mitogen-activated protein kinase and nuclear factor kappa light-chain enhancer of activated B cells (NFκB), leading to the activation of the inflammatory cascade and secretion of cytokines that are expressed in irreversibly inflamed pulps, including interleukin-6 (IL-6), interleukin-8 (IL-8), and tumor necrosis factor-α (TNF-α) [6,8,9,10]. It is believed that IL-6 promotes matrix degradation, IL-8 presence at the site of inflammation acts as a major attractant for neutrophils, while TNF-α induces further chemotaxis, neutrophil activation, and bone resorption [11,12,13]. To modulate the extent of inflammation, fibroblasts from the dental pulp secrete anti-inflammatory interleukin-10 (IL-10) to suppress the secretion of pro-inflammatory cytokines such as IL-6 and limit inflammation to the dentin-pulp interface by inhibiting NFκB pathway [14].

Inflammation and healing play a significant role in promoting hDPSCs differentiation to odontoblast-like cells [7]. Stimulating hDPSCs with a low quantity of TNF-α can promote the odontoblastic secretion of non-collagenous phosphorylated proteins in the early stage of reparative dentin process such as dentin phosphoprotein, dentin sialoproteins, dentin matrix protein 1, and osteocalcin [15]. However, stimulation with a high quantity of IL-6 and TNF-α decreased the differentiation and mineralization properties of hDPSCs [16]. In addition, an inverse relationship was noted between IL-8 levels with dentin sialoproteins levels in the dental pulp [17].

Management of pulpal disease associated with dental caries consists of removal of the etiologic agent and placing a material that will promote pulp healing and the development of a calcified dentin bridge. Various materials are used to reduce the inflammatory process and promote healing of the dental pulp. Calcium hydroxide was the first material to be used for this purpose, followed many years later by calcium silicate-based materials including mineral trioxide aggregate (MTA^®^), and Biodentine^TM^. Although MTA^®^ improved the dentin bridge quality in comparison to calcium hydroxide, inflammation was not significantly reduced with MTA^®^ compared to calcium hydroxide [18]. Furthermore, MTA promoted macrophage activation, initiating the synthesis of pro-inflammatory cytokines [19]. In fact, calcium silicate materials elevated pro-inflammatory cytokine levels, for example, MTA^®^ increased the levels of IL-8, IL-6, and TNF-α, while Biodentine^TM^ increased the levels of IL-6 [19,20,21]. Thus, the induced dentin bridge might be enhanced by ameliorating the levels of pro-inflammatory cytokines due to the inverse relationship between levels of pro-inflammatory cytokines and dentinogenic markers [16,17].

Polydatin, also known as piceid is a monocrystalline compound and a resveratrol precursor, considered a traditional polyphenolic Chinese medicine compound that has been naturally extracted from Japanese knotweed (*Fallopia japonica*) and found in multiple other plants [22]. Polydatin has been found to possess osteogenic differentiation properties as well as promoting odontogenic differentiation of hDPSCs by upregulating odontogenic markers and size and number of mineralized nodules [22,23]. In addition, polydatin is known to have anti-inflammatory properties among other beneficial effects such as antioxidant, anti-aging, antimicrobial, hepatoprotective, and neuroprotective effects [9,22,24,25,26,27]. In fact, treatment of pulpal and non-pulpal cells with polydatin or resveratrol decreased IL-8 and IL-6 levels as detected by enzyme-linked immunosorbent assay (ELISA) and reverse transcription–quantitative polymerase chain reaction (RT-qPCR) [20,28,29,30,31].

The anti-inflammatory mechanism of polydatin and its derivative resveratrol has been linked to suppressed activation of NFκB by diminishing its activator IκB kinase, thus delaying the phosphorylation of NF-ĸB inhibitor IκBα [29,31,32]. In addition, polydatin and resveratrol decreased the phosphorylation levels of c-Jun N-terminal kinase and P38 of the mitogen-activated protein kinase pathway, which is linked to IL-6 and IL-8 transcription [31,33,34,35].

A natural compound that can promote odontogenic differentiation while reducing the inflammation of the dental pulp could prove advantageous in the management of dental caries-related pulp disease. Therefore, the aim of this study was to assess the effect of polydatin on the viability and inflammatory cytokines expression levels associated with inflammation of the pulp in LTA-stimulated hDPSCs. The null hypothesis of this study stated that polydatin has no modulatory inflammatory effect on LTA-stimulated hDPSCs.

## 2. Materials and Methods

### 2.1. Overview of the Methodology

The methodology of this in vitro study evaluated the viability of LTA-stimulated hDPSCs with no polydatin, and LTA-stimulated hDPSCs treated with 1 µM, 0.1 µM, and 0.01 µM concentrations of polydatin. Followed by quantifying the levels of pro- and anti-inflammatory cytokines, IL-6, IL-8, IL-10, and TNF-α, by RT-qPCR and ELISA. All of the experiments were performed by one experienced investigator.

### 2.2. Cells and Cell-Culture Conditions

#### Cell Expansion

Commercially available Axol Bioscience LTD human dental-pulp stem cells isolated from a healthy donor were used (catalog number ax3901, Axol, Cambridge, UK). Supplemented alpha-modified minimum essential medium (α-MEM) (Thermo Fisher Scientific, Waltham, MA, USA) was used to culture the cells. The α-MEM supplements used were as follows; 10% fetal bovine serum, 1% penicillin/streptomycin, and 1% MEM non-essential amino acid solution (Thermo Fisher Scientific). Cells were cultured in an incubator at 37 °C under 95% humidity and 5% CO_2_ conditions using 25 cm^2^, 75 cm^2^, and 175 cm^2^ flasks until the desired passage was reached. After reaching 90% of cellular confluency, the cells were transferred to the desired test plates to be investigated in their respective assays. All experiments used cells in passages three to five. The media was changed every two to three days of culturing.

### 2.3. Materials Preparation

#### 2.3.1. Polydatin Preparation

Polydatin material, purchased commercially (Shandong Zhi Shang Chemical Ltd., Jinan, China, CAS 27208-80-6), was used. The polydatin material for this experiment was prepared by dissolving it in 0.1% dimethyl sulfoxide (DMSO) before adding it to the culture medium in the desired concentration as per the instructions of the manufacturer. Three concentrations of polydatin were prepared, namely, 1 µM, 0.1 µM, and 0.01 µM.

#### 2.3.2. Lipoteichoic Acid Stimulation of Dental-Pulp Stem Cells

A purified LTA, TLR-2 agonist, which is an endotoxin and virulence factor extracted from Gram-positive bacteria *Staphylococcus aureus* (*S. aureus*) was purchased from (InvivoGen, San Diego, CA, USA, CAS 56411-57-5) aliquoted to 10 µg according to the manufacturer’s protocol, was used to induce hDPSCs for two hours, then treated with 1 µM, 0.1 µM, and 0.01 µM polydatin, while the control group had the same medium without polydatin.

### 2.4. Cellular Viability of hDPSCs Exposed to Polydatin

#### AlamarBlue^TM^ Cell Viability Assay

Polydatin effect on the viability of LTA-stimulated hDPCs was assessed at 1 µM, 0.1 µM, and 0.01 µM concentrations. AlamarBlue^TM^, a non-toxic assay, was used on days 1, 3, and 7 (Thermo Fisher Scientific). Ninety-six well plates were used to seed the cells at 5 × 10^3^ cells per well and kept in an incubator at 37 °C under 95% humidity and 5% CO_2_ conditions. The media was removed at each experiment endpoint and 10% of alamarBlue^TM^ reagent in supplemented α-MEM was added to each well. SpectraMax^®^ M5/M5e Multimode Plate Reader (Molecular Devices, San Jose, CA, USA), at an excitation of 530 nm and an emission of 590 nm, was used to measure the fluorescence based on the redox reaction of the AlamarBlue^TM^ active ingredient resazurin, which is weakly fluorescent and is transformed by metabolically active cells to the highly fluorescent resorufin. SoftMax^®^ Pro 6 Microplate Data Acquisition and Analysis Software (Molecular Devices) was used to acquire data. The experiment was duplicated for each time point, with 10 wells for each group [36].

### 2.5. Levels of Cytokines in LTA-Stimulated Dental-Pulp Stem Cells

#### 2.5.1. Reverse Transcription—Quantitative Polymerase Chain Reaction (RT-qPCR)

Dental-pulp stem cells were seeded in a 6-well plate in a supplemented α-MEM with a density of 5 × 10^5^ cells/well. On the second day, cells were stimulated for two hours with 10 µg LTA and simultaneously treated with different concentrations of polydatin at 1 µM, 0.1 µM, and 0.01 µM. RNeasy mini kit (RNeasy; Qiagen, Hilden, Germany) was used for RNA isolation following the addition of lysis buffer in each well. Nanodrop spectrophotometer (Nanodrop 2000, Thermo Fisher Scientific) was used to quantify RNA concentration. Next, cDNA was synthesized from extracted RNA using a high-capacity cDNA reverse transcription kit (Thermo Fisher Scientific) and a multigene cycler (Labnet International, Inc., Edison, NJ, USA). Thereafter, the expression of messenger RNA (mRNA) was analyzed using Fast SYBR^TM^ Green PCR Master Mix (Thermo Fisher Scientific). Thermal cycler parameters for denaturation were set at 95 °C for 1 s, annealing and extension temperatures were set at 60 °C for 20 s, and the cycles were repeated for 40 cycles. Table 1 depicts IL-6, IL-8, IL-10, and TNF-α sense and anti-sense primer sequences (Oligo^TM^, Seoul, Republic of Korea) [37]. Groups were run in duplicated experiments, and amplification was run for 40 cycles. Glyceraldehyde-3-phosphate dehydrogenase (GAPDH) was used to normalize the cycle threshold (Ct) values that were obtained. Amplification cycles and cycle threshold (Ct) values were obtained and normalized to the endogenous control values. The data were normalized following the delta–delta Ct method (2^−ΔΔ CT^) using the endogenous reference (GAPDH) [38].

#### 2.5.2. Enzyme-Linked Immunosorbent Assay

LTA, a TLR-2 agonist, was used to stimulate hDPSCs for four hours in a 96-well plate, for the detection of the pro-and anti-inflammatory cytokines, IL-6, IL-8, IL-10, and TNF-α. Supernatants of all of the tested cytokines were collected following the standard protocol of ELISA [6]. Collected samples were centrifuged then aliquoted and preserved in −80 °C.

The manufacturer’s protocol for IL-6, IL-8, IL-10, and TNF-α was followed for reconstituting the standards of the studied cytokines (Thermo Fisher Scientific, KAC1261; CAS EHIL10; KAC126; BMS223-4). Antibody-coated wells received standards and samples of IL-6, IL-8, IL-10, and TNF-α to allow specific antigen–antibody binding for their respective coated adsorbed antibodies. Thereafter, the reagent solution was removed, and the plates were washed. The antigens bound to the plate via the primary immobilized adsorbed antibody were bound to the added biotin conjugate antibody (detection antibody). Subsequent aspirations and wash steps were followed. Finally, biotin conjugate plates had streptavidin-HRP solution added, plates were washed to eliminate unbound antibodies. The chromogen substrate was added to the wells and left until the desired color change was achieved, then the reaction was terminated using a stop solution. SpectraMax^®^ M5/M5e Multimode Plate Reader (Molecular Devices) was used to read absorbance at 450 nm.

SoftMax^®^ Pro 6 Microplate Data Acquisition and Analysis Software (Molecular Devices) was used to acquire the data. The experiments were performed in duplicate for each group. A standard curve was generated using GraphPad Prism 10 software (GraphPad Software, San Diego, CA, USA).

### 2.6. Statistical Analysis

Results were expressed as the mean ± standard deviation where the control group was used for normalization of the data. Shapiro–Wilk test was used to test the normality of the distribution of all of the acquired data. RT-qPCR and ELISA data were analyzed using the one-way ANOVA parametric test, then the equality of variance was verified using the Levene test, and finally Tukey post hoc multiple-comparison test was used. When the equality of variance assumption was violated, the Welch ANOVA test was used with Games–Howell post hoc multiple-comparison test. A non-parametric test (Kruskal–Wallis) was performed for the viability assay data that did not pass the Shapiro–Wilk normality test. Data was then analyzed by a Dunn–Bonferroni pairwise multiple-comparison test to compare values between groups of the viability assay. The statistical significance level was set at *p*-value < 0.05. IBM Statistical Package for the Social Sciences (SPSS) Statistics software (version 29) was used for statistical analysis. GraphPad Prism 10 software (GraphPad Software) was used to design graphs.

## 3. Results

### 3.1. Viability of Stimulated Dental-Pulp Stem Cells

**Figure 1:**
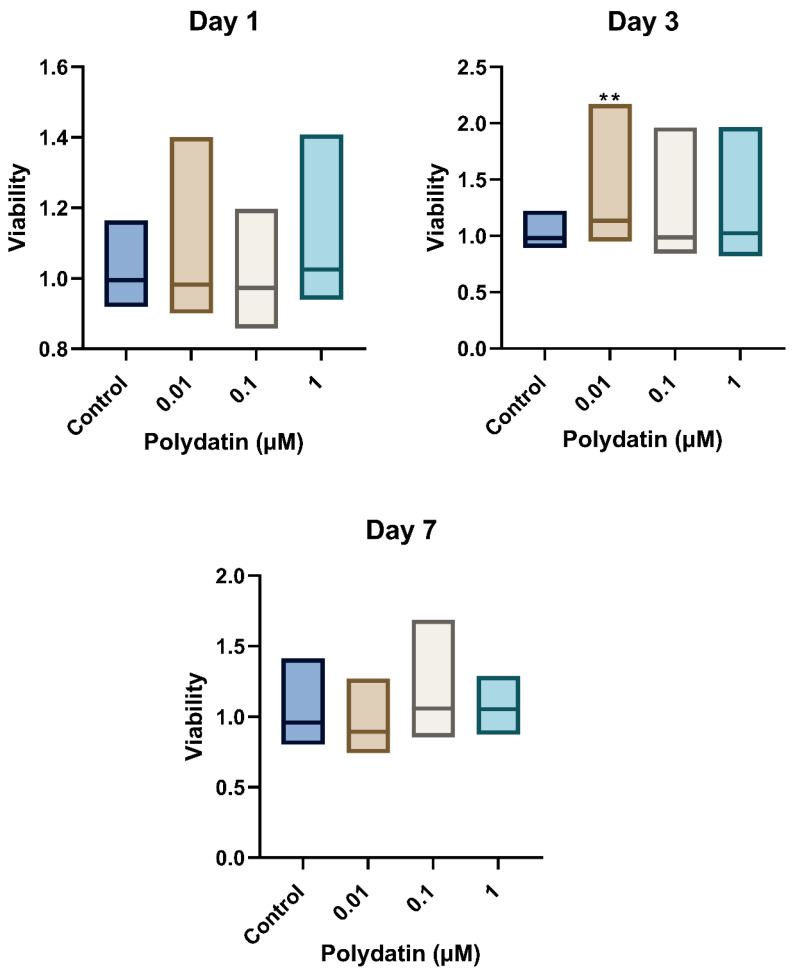
Viability of LTA-stimulated hDPSCs on days 1, 3, and 7. Viability is expressed in optical density (O.D.) at an excitation of 530 nm and an emission of 590 nm with or without treatment with polydatin. Data is presented in a floating bar graph with the median. The experiment was duplicated for each time point. A Kruskal–Wallis non-parametric test followed by a Dunn–Bonferroni pairwise multiple-comparison post hoc test were used to analyze the data. ** indicates a statistically significant difference compared to the control group (** *p* < 0.01).

Using the non-parametric Kruskal–Wallis test, polydatin affected the viability of LTA-stimulated hDPSCs (*p* < 0.01). The Dunn–Bonferroni pairwise multiple-comparison post hoc test indicated that 0.01 µM polydatin on day 3 (Figure 1) produced a significant increase in cellular viability compared to the control group (*p* < 0.01).

### 3.2. Effect of Polydatin Treatment on Messenger RNA Levels

One-way ANOVA parametric test showed a statistically significant reduction in the mRNA levels of IL-6 (*p* < 0.01), IL-8 (*p* < 0.05), and TNF-α (*p* < 0.01), while showing a significant increase in the IL-10 mRNA levels (*p* < 0.05) after polydatin treatment.

**Figure 2:**
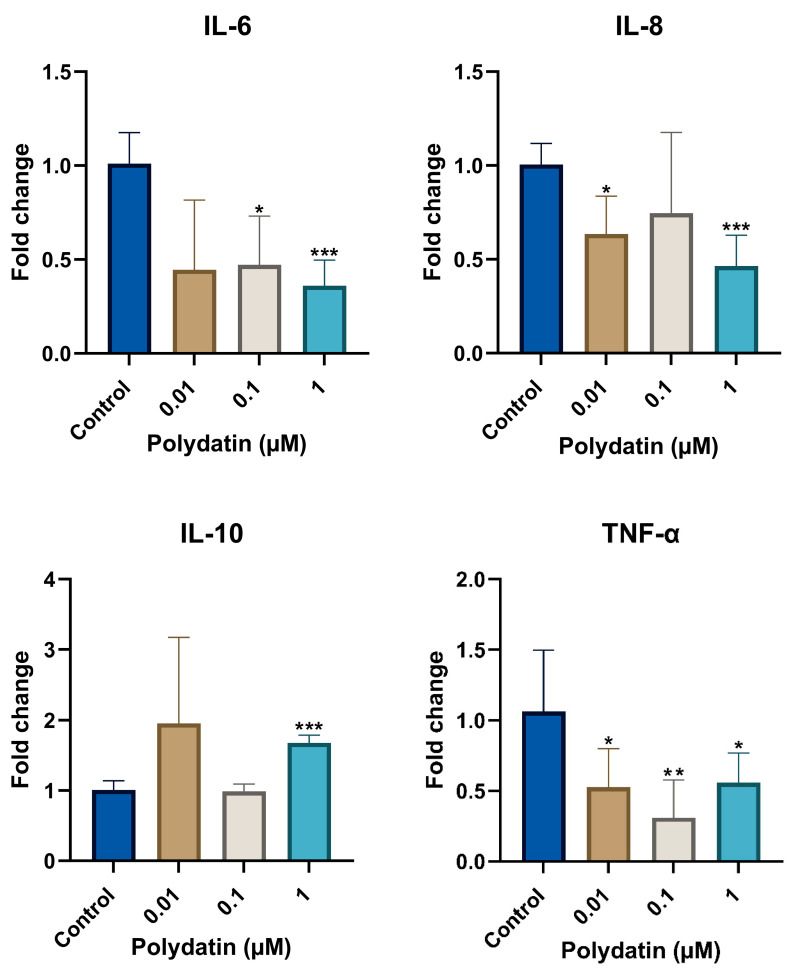
Fold change in LTA-stimulated hDPSCs mRNA levels measured by RT-qPCR of IL-6, IL-8, IL-10, and TNF-α. Levels of mRNA are measured by RT-qPCR of IL-6, IL-8, IL-10, and TNF-α of hDPSCs after 2 h of LTA stimulation and polydatin treatment. Data is presented in a column graph with mean ± SD. Groups were run in duplicated experiments. A one-way ANOVA parametric test followed by a Games–Howell post hoc test were used to analyze the data. * indicates a statistically significant difference in comparison with the control group (*** *p* < 0.001, ** *p* < 0.01, * *p* < 0.05).

Using the Games–Howell post hoc test, polydatin treatment of LTA-stimulated hDPSCs significantly reduced mRNA levels of IL-6 with 0.1 µM (*p* < 0.05), and 1 µM (*p* < 0.001). Similarly, IL-8 mRNA levels displayed a significant reduction with 0.01 µM (*p* < 0.05) and 1 µM (*p* < 0.001). In addition, TNF-α was significantly reduced with all polydatin concentrations at 0.01 µM (*p* < 0.05), 0.1 µM (*p* < 0.01), and 1 µM (*p* < 0.05), while IL-10 showed a significant increase with 1 µM (*p* < 0.001), as shown in Figure 2.

### 3.3. Effect of Polydatin Treatment on Cytokine Levels

**Figure 3:**
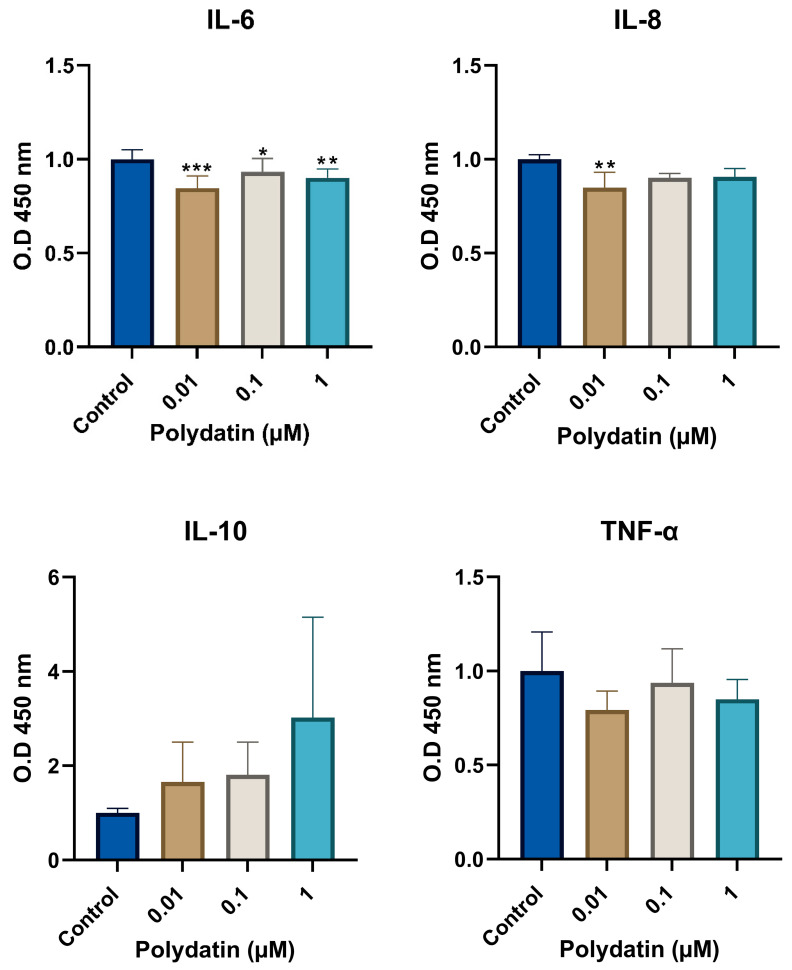
Cytokine levels as measured by enzyme-linked immunosorbent assay (ELISA) of IL-6, IL-8, IL-10, and TNF-α. Cytokine levels expressed in optical density (O.D.) with an absorbance at 450 nm read by a SpectraMax^®^ M5/M5e Multimode Plate Reader of IL-6, IL-8, IL-10, and TNF-α of hDPSCs after 4 h of LTA stimulation and polydatin treatment. Data is presented in a column graph with mean ± SD. The experiments were performed in duplicate for each group. A one-way ANOVA parametric test followed by a Games–Howell post hoc test were used to analyze the data. * indicates a statistically significant difference compared to the control group (*** *p* < 0.001, ** *p* < 0.01, * *p* < 0.05).

A statistically significant decrease in the IL-6 (*p* < 0.001) and IL-8 (*p* < 0.01) levels, as demonstrated by the one-way ANOVA test, was observed after polydatin treatment, as shown in Figure 3.

Using the Games–Howell post hoc test, IL-6 cytokine levels were significantly decreased with all concentrations of polydatin with 0.01 µM (*p* < 0.001), 0.1 µM (*p* < 0.05), and 1 µM (*p* < 0.01). In addition, IL-8 cytokine levels were reduced in response to polydatin, with 0.01 µM being statistically significant (*p* < 0.01). On the other hand, TNF-α levels exhibited a non-statistically significant decrease with polydatin treatment in all concentrations, with the highest reduction noted with 0.01 µM. Similarly, IL-10 levels were elevated after polydatin treatment; however, the increase was not statistically significant.

## 4. Discussion

Inflammation in the dental pulp can be initiated by hDPSCs as well as several other cell types in response to invading microbes and microbial products [6]. In dental caries, many Gram-positive bacterial genera, such as *Streptococcus*, *Olsenella*, *Propionibacterium*, and *Lactobacillus*, are involved in symptomatic irreversible pulpitis [39]. LTA is a virulence factor for Gram-positive bacteria functioning as a ligand that binds to toll-like receptor-2 located at the cell membrane to act as an innate immune response and release cytokines associated with inflammation, for example, IL-6, IL-8, IL-10, and TNF-α [6,8,40]. Dental-pulp stem cells can promote pulpal healing and odontogenic differentiation by ameliorating the process of inflammation [16]. Hence, therapeutic agents that could decrease cytokines associated with inflammation such as IL-8 and IL-6 can be linked to increased differentiation and fold increase in odontogenic markers [16,17]. This is attributed to the inverse relationship between the levels of pro-inflammatory cytokines and odontogenic markers [17]. In fact, IL-6 and TNF-α stimulated DPSCs significantly inhibited osteogenic differentiation with a lack of osteogenic colonies, calcium deposits, and directed the cells to a more fibrous morphology [16]. In the current study, adding polydatin to LTA-stimulated hDPSCs led to a reduction in IL-6, IL-8, and TNF-α levels which are cytokines associated with promoting inflammation, while producing an increase in IL-10 levels which is a cytokine linked to suppressing inflammation.

The observed effect of polydatin treatment manifested in combating inflammation in the current study, namely a decrease in IL-6, IL-8, and TNF-α levels while showing an increase in IL-10 levels, is in agreement with previous studies using different cell types such as LPS and LTA-stimulated macrophages and *S. aureus*-stimulated mouse mammary epithelial cells, and in vivo experimental models [28,34,40]. The underlying modulation of the inflammation effect, which decreased cytokines that are considered pro-inflammatory and increased the cytokine that is considered anti-inflammatory, was attributed to the suppression and reduction in components activating inflammatory pathways of NFκB and mitogen-activated protein kinase [28,29,31,32,33,34,35]. Treatment of LPS-stimulated macrophages with polydatin decreased the phosphorylation levels of p65 protein kinase, blocking the activation of NFκB pathway [28]. In addition, polydatin treatment on *S. aureus*-stimulated mouse mammary epithelial cells prevented TLR-2 expression and suppressed the activation of NFκB and mitogen-activated protein kinase [34]. Polydatin’s protective effects in modulating the inflammatory process include the reported reduction in neutrophil infiltration in an in vivo animal model and maintaining the integrity of the epithelial barrier after polydatin treatment [28].

Several materials have aimed to heal inflamed pulp and promote calcific formation at the pulp–dentin interface by reducing inflammation, such as calcium hydroxide and calcium silicate materials. Although calcium silicate materials have enhanced the quality of the dentin bridge compared to calcium hydroxide, they did not contribute positively to modulating the inflammatory process in the inflamed pulp. A noticeable increase in pro-inflammatory cytokines was noted with calcium silicates, elevating cytokine levels of IL-8, IL-6, and TNF-α [18,19,20,21]. That may act as a contributing factor to the quality of the induced dentin bridge [16,17]. This is probably related to the reported polydatin’s protective effects on heat-stressed keratinocytes and LPS-stimulated murine macrophage cells, decreasing IL-6, IL-8, and TNF-α, and promoting IL-10 cytokine levels [28,34].

Based on the findings of the current study, the observed polydatin-related modulation of inflammatory cytokines associated with the inflamed dental pulp, IL-6, IL-8, IL-10, and TNF-α, suggests a beneficial role of polydatin in the healing of damaged dental pulp. This cytokine modulation observed in the current study, together with the reported osteogenic/odontogenic differentiation effect of polydatin [23], makes this material a good candidate for the management of irreversibly damaged pulp. Despite the encouraging results shown in this work, it must be remembered that these experiments were conducted in a controlled in vitro environment that lacks the complexity of the real dental pulp with its multiple cell types, immune mechanisms, and pathogen interactions [41,42]. In addition, one virulence factor, Gram-positive LTA, was used in comparison with multiple pathogens with their metabolites and toxins that are present in the oral environment. LTA extracted from cariogenic bacteria such as *Streptococcus mutans* can replicate the clinical conditions of the inflamed pulp more closely. However, the current study used commercially prepared *S. aureus* LTA, predominantly found on the skin and in the upper respiratory tract, because it has more potent immunostimulatory activity than LTA purified from *Streptococcus mutans* and is mostly used with dental-pulp cells to stimulate inflammation [8,43,44,45,46,47]. Furthermore, hDPSCs were obtained commercially from a single source; thereby, the effects of donor age, health status, genetic background, and stage of tooth development at harvest might affect the observed results. Therefore, the promising in vitro findings of this research must be confirmed with an in vivo model that replicates more closely the clinical situation with its complex environment.

Thus, the beneficial influence of polydatin on inflamed pulp tissues of the tooth caused by multiple species of cariogenic bacteria should be investigated in an in vivo animal model, followed by clinical studies to further study the properties of polydatin in the complex environment of the dental pulp before it is considered as part of the management of teeth that require pulp healing and formation of a hard tissue bridge.

## 5. Conclusions

In conclusion, adding polydatin material to LTA-stimulated hDPSCs modulated the expression of the inflammatory mediators by mainly suppressing the levels of cytokines that are prevalent in inflamed pulps and considered pro-inflammatory, such as IL-6, IL-8, and TNF-α, while promoting a cytokine that is considered anti-inflammatory, namely, IL-10, suggesting a beneficial role in pulp healing.

## Figures and Tables

**Table 1:** GAPDH, IL-6, IL-8, IL-10, and TNF-α sense and anti-sense primer sequences (forward and reverse). Reprinted from [37].

Gene	Sequence (5′-3′)
GAPDH	Sense (forward primer) 5′-CTGGTAAAGTGGATATTGTTGCCAT-3′ Antisense (reverse primer) 5′-TGGAATCATATTGGAACATGTAAACC-3′
IL-6	Sense (forward primer) 5′-GCCCAGCTATGAACTCCTTCT-3′ Antisense (reverse primer) 5′-GAAGGCAGCAGGCAACAC-3′
IL-8	Sense (forward primer) 5′-GGCACAAACTTTCAGAGA CAG-3′ Antisense (reverse primer) 5′-ACACAGAGCTGCAGAAATCAGG-3′
IL-10	Sense (forward primer) 5′-TGAGCTTCTCTGTGAACGATTTA-3′ Antisense (reverse primer) 5′-GTCACCCTATGGAAACAGCTTA-3′
TNF-α	Sense (forward primer) 5′-GAGGCCAAGCCCTGGTATG-3′ Antisense (reverse primer) 5′-CGGGCCGATTGATCTCAGC-3′

Abbreviations: GAPDH, Glyceraldehyde-3-phosphate dehydrogenase; IL-6, interleukin-6; IL-8, interleukin-8; IL-10, interleukin-10; TNF-α, tumor necrosis factor α.

## Data Availability

The original contributions presented in the study are included in the article, further inquiries can be directed to the corresponding author.

## Abbreviations

The following abbreviations are used in this manuscript:

LTA	Lipoteichoic acid
hDPSCs	Human dental-pulp stem cells
ELISA	Enzyme-linked immunosorbent assay
IL-6	Interleukin-6
IL-8	Interleukin-8
IL-10	Interleukin-10
TNF-α	Tumor necrosis factor α
NF-ĸB	Nuclear factor kappa light chain enhancer of activated B cells
MTA^®^	Mineral trioxide aggregate^®^
α-MEM	Alpha-modified minimum essential medium
DMSO	Dimethyl sulfoxide
*Staphylococcus aureus*	*S. aureus*
RT-qPCR	Reverse transcription-quantitative polymerase chain reaction
GAPDH	Glyceraldehyde-3-phosphate dehydrogenase
